# Instruments used to assess language in low‐educated individuals with mild cognitive impairment: a systematic review

**DOI:** 10.1002/alz.087877

**Published:** 2025-01-03

**Authors:** Emily Viega Alves, Bárbara Costa Beber, Raphael Machado Castilhos

**Affiliations:** ^1^ Federal University of Rio Grande do Sul, Porto Alegre, Rio Grande do Sul Brazil; ^2^ Universidade Federal de Ciências da Saúde de Porto Alegre (UFCSPA), Porto Alegre, RS Brazil; ^3^ Universidade Federal do Rio Grande do Sul, Porto Alegre, Rio Grande do Sul Brazil; ^4^ Hospital de Clínicas de Porto Alegre, Porto Alegre, Rio Grande do Sul Brazil

## Abstract

**Background:**

Mild Cognitive Impairment (MCI) is a cognitive decline that is greater than expected for an individual’s age and level of education, but which does not interfere with functionality. It may be divided into amnestic (when it predominantly affects memory) and non‐amnestic (other cognitive domains). One of the cognitive functions that may be affected by MCI is language. As this is a function closely linked to education, it is essential to understand how it is assessed in people with low levels of schooling in different populations, such as those with cognitive impairment. Therefore, this study aimed to perform a systematic review of measures of language in low‐educated individuals with MCI.

**Method:**

This is a systematic review of the literature. The search was performed in four databases: PubMed, Embase, LILACS, and Scielo, using MeSH descriptors related to MCI with low education and in which there was a description of tests used. We included papers that assessed language domain in individuals with MCI. The PRISMA guideline was adopted to better conduct the review.

**Results:**

The search strategy initially resulted in 2978 articles. After excluding duplicates and applying the eligibility criteria, 23 papers were selected for full‐text reading and 8 were included for analysis. The tools used for language assessment were not standardized, ranging from isolated tasks to subtests of larger instruments and complete batteries. The most common instrument was the Boston Diagnostic Aphasia Examination (25%), which was applied completely in one study and partially in another. The most commonly tested language skills were verbal fluency assessed in 75% of the works and naming, present in 50% of the studies. Skills such as reading and writing were little explored, possibly because educational issues impact on the possibility of reliably assessing these abilities.

**Conclusion:**

There is no consensus and no specific instruments for assessing language in low‐educated individuals with MCI. In addition, few language skills are tested in this population, which means there is a need to create better‐validated tests designed for this public.

